# Medical Students’ and Radiology Technician Trainees’ eHealth Literacy and Hygiene Awareness—Asynchronous and Synchronous Digital Hand Hygiene Training in a Single-Center Trial

**DOI:** 10.3390/healthcare11101475

**Published:** 2023-05-18

**Authors:** Christian Kühnel, Sarah Salomo, Helena Pagiatakis, Jutta Hübner, Philipp Seifert, Martin Freesmeyer, Falk Gühne

**Affiliations:** 1 Clinic of Nuclear Medicine, Jena University Hospital, Am Klinikum 1, 07747 Jena, Germany; 2 Clinic for Internal Medicine II, Jena University Hospital, Am Klinikum 1, 07747 Jena, Germany

**Keywords:** ehealth, ehealth literacy, eHEALS, hand hygiene, health education, hospital care

## Abstract

Next to the known nosocomial infections, the COVID-19 pandemic was an example for the need for the immediate implementation of functioning hygiene concepts and knowledge transfer. The aim of this study was to evaluate the self-assessment of ehealth literacy in terms of finding, using and critically evaluating health information and theoretical and practical hygiene awareness on a voluntary participation basis at the Jena University Hospital in 2022. The well-established and validated eHEALS and WHO questionnaire on hand hygiene (HH) knowledge for healthcare workers was completely filled by 204 participants (191 medical students; 13 healthcare trainees). In a second step, after the questionnaire, 77 participants completed additional asynchronous, digitally guided self-training using DesiCoach 2Go. In the end, a synchronous hand disinfection was carried out in the hospital using Visirub, by separating it into a group without (*n* = 191; with and without HH questionnaire) and a group with (*n* = 31; with HH questionnaire) previously completed self-training. For the eHL, the respondents tended to have a positive self-assessment of finding, using and critically evaluating health information. The voluntary participants of the practical hand disinfection who had received self-training were able to achieve significantly better results (*p* = 0.0047), resulting in fewer wetting gaps in a subsequent performance with Visirub than those who had not received digital self-training. The survey showed that healthcare-related participants belonging to the “digital native” generation have above-average knowledge on HH and profit by digitally guided self-training.

## 1. Introduction

Nosocomial infections are infections that patients can acquire in hospitals or other healthcare facilities. They pose a serious threat to hospital hygiene because they are significant causes of illness or death and can also increase the overall cost of healthcare [1,2]. In addition to transmission during invasive procedures such as surgery, catheterization and ventilation, staff transmission and equipment transmission are also important. Specifically, during contact between hospital staff or medical equipment and patients, pathogens can be transmitted from one to another if proper hygiene measures are not taken [3]. For these reasons, it is essential that hospitals implement, train and monitor strict hygiene measures to minimize the spread of nosocomial infections [4].

In the specific case of a new severe respiratory syndrome, namely SARS-CoV-2, which emerged in Wuhan, China, between December 2019 and January 2020 and then spread rapidly worldwide, hygiene measures have taken on almost-unprecedented global importance [5]. This virus has been declared a public health emergency by the World Health Organization (WHO) [6]. Infection with SARS-CoV-2 has a wide spectrum of symptoms [7,8,9,10,11]. Identifying rapidly progressing patients remains a challenge, and various treatment options (e.g., antivirals, nutritional supplements, etc.) have been proposed [12,13]. SARS-CoV-2 certainly has had a major social and psychological impact on the general population and their behavior [14,15,16,17].

Normally, in view of the human-caused infection chains for nosocomial infections, hand disinfection is the most efficient instrument to reduce or avoid them if it is carried out correctly and in a prescribed manner [18]. During the SARS-CoV-2 pandemic, some behaviors changed and came to be perceived as normal, which prevented infection [19,20]:

- Carrying disinfectant on us.
- Wearing mouth-nose protection (MNP).
- Using our mobile phones as sources of information and digital identification tools.

Furthermore, other aspects of daily life have changed: instead of shaking hands, the elbow or the fist was used for greetings [21]. While people once went to work if they had minor symptoms, today they stay at home or use the option of remote working or a home office as a health-promoting work arrangement [22].

In this context, increasing digitalization, such as the introduction of the digital vaccination record (e.g., apps like CovPass), ehealth literacy (eHL) (i.e., digital health skills) and hygiene awareness, is currently crucial. Norman and Skinner developed the well-known Lily model for eHL in 2006, which was based on scientific publications of the time and recommended six essential components [23,24,25]. These six components are required to have the various skills, knowledge and competencies that should significantly influence behavioral patterns.

- Analytical skills:
  ○ Information literacy (dealing with information).
  ○ Media literacy (handling new media).
  ○ Traditional literacy (education level, reading, spelling and everyday math skills).

- Context-specific skills:
  ○ General health literacy (biopsychosocial understanding of health).
  ○ Computer literacy (digital competence).
  ○ Use of scientific information sources (ability to understand, evaluate and interpret scientific findings).

Regarding students and trainees in healthcare, it can therefore be assumed that the perception of proper behavior in a professional environment has changed [26]. Possible reasons for this include that the period of study and training, during the SARS-CoV-2 pandemic, including synchronous or asynchronous distance teaching, featured increased hygiene measures and that digital contact data collection at face-to-face events has had a sensitizing influence. Of course, there is also the suspicion that social conventions contribute to the fact that students and trainees in healthcare and hospitals, as “digital natives” (born after 1980), perceive development as normal [27].

The aim of this study was to find out how medical students (MSs) and radiology technician trainees (RTs) at Jena University Hospital assess their eHL, taking into account internal consistency and reliability. Of the seven questionnaires currently used in the literature to measure self-assessments of ehealth, the eHEALS questionnaire is the most widely used [28]. Furthermore, the knowledge about hand hygiene (HH) and the performance of hand disinfection by the participants should be evaluated. The why, how and when of HH was defined by the WHO in 2009, which is why the WHO questionnaire might be the best choice for use [29]. In addition, how well the participants disinfect themselves and how well this is performed, especially by those who have completed digital asynchronous self-training in the past, should be examined.

## 2. Materials and Methods

**Figure 1:**
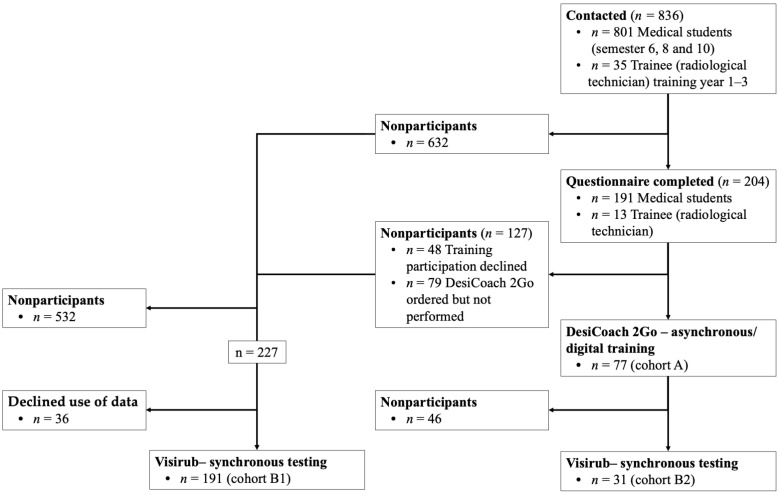
Flowchart for study participation.

The present study is separated into three sections: questionnaire, asynchronous digital hand HH self-training and synchronous hand disinfection (Figure 1). After the participants were contacted, they voluntarily participated in a survey using two combined questionnaires. At the end of the questionnaire, the participants had the opportunity to agree to receive an asynchronous digital training kit for hand disinfection training in accordance with data protection. The last step included the synchronous control of the HH disinfection, including wetting gaps during an internship inside the clinic for nuclear medicine. For the last step, participants were asked whether the DC training kit had already been received *and* used or whether the results of the synchronous results recording could be used.

The questionnaire investigations in this study were limited to MSs of the Friedrich Schiller University Jena (FSU) in the sixth, eighth and tenth semesters and RTs of the State Vocational School for Health and Social Affairs Jena, between the first and third years of training. Both groups were taught relevant aspects of hygiene and HH in the first year. The MSs completed a mandatory hygienic hand disinfection course in the first or second semester, in the university’s Skills Lab (training facility for practical skills). The RT attended a complete hygiene course in the first year of training, including practical application.

### 2.1. Questionnaire

For the survey on ehealth literacy (eHL) and hygiene awareness, a combined questionnaire consisting of the German translation of the eHEALS questionnaire (8 items) and the WHO HH Knowledge Questionnaire for Health-Care Workers (5 items for sociodemographic evaluation and 25 items for knowledge evaluation) was used [30,31,32].

Two additional items (usefulness and importance) were recommended by Norman and Skinner to the eHEALS questionnaire to capture consumers’ interest in using ehealth in general [31]. The additional recommended items were not asked with a 5-point Likert scale (strongly agree, agree, undecided, disagree, strongly disagree); instead, they were asked with a 4-point Likert scale (very useful/important, useful/important, not useful/important, not useful/important at all) to avoid abstentions or the neutral center.

To the WHO questionnaire, two additional items were added to capture the delivery of hygiene training. For the questionnaire section on HH, each item was assigned a point for evaluating the participants’ level of knowledge. The survey was conducted exclusively in digital form (using www.soscisurvey.de, accessed on 1 August 2022, which completely meets the data protection standard) with a survey period of 2 months (June and July 2022). At the end of the questionnaire, the surveyed participants had the opportunity to register for self-training (cohort A) with the hand disinfectant DesiCoach 2Go (DC) (Heyfair GmbH, Jena, Germany). After providing the address data, the DC kit was sent to the home of all interested volunteers by mail.

### 2.2. DesiCoach 2Go (Cohort A)

**Figure 2:**
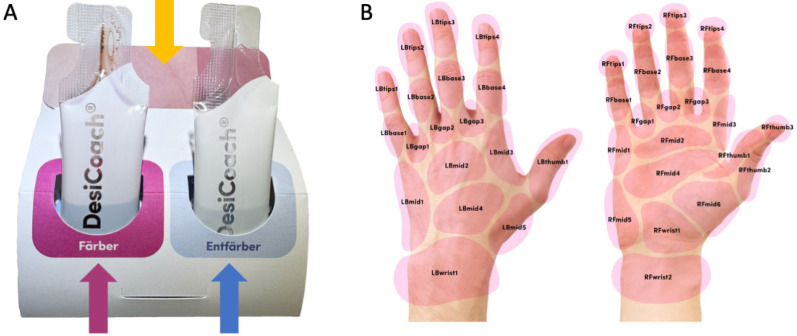
(**A**) DesiCoach 2GO packaging, including staining (pink arrow), decolorizing stick pack (blue arrow) solution, color card (orange arrow) and QR code manual (not shown). (**B**) Area mapping selecting in the web app.

At the end of the questionnaire, voluntary consent was requested and obtained for participants to receive DC training kits for asynchronous self-training, delivered by parcel carrier. DC is the first training kit in the world for hand disinfection that can be carried out independently and makes mistakes in disinfection visible without additional hardware. The DC kit (Figure 2A) consists of a staining solution (made of plant extracts that stain the hands reddish after rubbing), decolorizing solution, a color card (for evaluating whether the correct amount of disinfectant was used) and digital training instructions via QR code. The QR code links to the data collection page, including all necessary information (www.hygiene-training.com; accessed on 18 March 2023; access code for demo is “DEMO”).

However, the coloring happens only gradually, so the participants saw the success of the hand disinfection after only nearly 1 min. At the end of the waiting time, the user can confirm what color intensity has been achieved (with the color card shown in Figure 2A, in three levels: perfect, too little and far too little) and mark any wetting gaps on a web-based hand area map (Figure 2B). Normally, the training includes a second step of decolorizing and an additional evaluation of this step. For consistency and comparability with Visirub, the third step, the decolorizing, was not recorded and evaluated. The presentation of the results via the analytic portal (access given to the hygiene officer or representatives) is limited and offers simple visual information about the number of trainings used, the efficacy (amount of disinfectant used compared to the degree of staining) and the degree of wetting in the form of a heatmap presentation. The front side, i.e., the palm (*n* = 22) and the back side of the hand (*n* = 18) of both hands, is divided into 40 areas each (total *n* = 80 areas) (Figure 2B). By using the number of possible wetting gaps, the evaluation page of DC calculates from the absolute numbers the relative results on the participant group. On the basis of the raw data, it was possible to separately examine the number of wetting gaps on the front side and those on the back side of the hand for cohort A.

### 2.3. Visirub (Cohort B1 and B2)

During a practical seminar at the Department of Nuclear Medicine, 258 participants were guided through three wards in small groups (*n* = 191 were assigned to B1; *n* = 31 were assigned to B2; and *n* = 36 declined to participate or to have their data used). Owing to COVID-19, special requirements were imposed on face-to-face events. For teaching events in areas of patient care, students had to sign a form (“Declaration on classroom teaching during the COVID-19 pandemic”) confirming their attendance at a presentation (29 slides) on the topic of infection prevention in hospitals and HH (provided by the Institute for Infection Medicine and Hospital Hygiene of Jena University Hospital).

**Figure 3:**
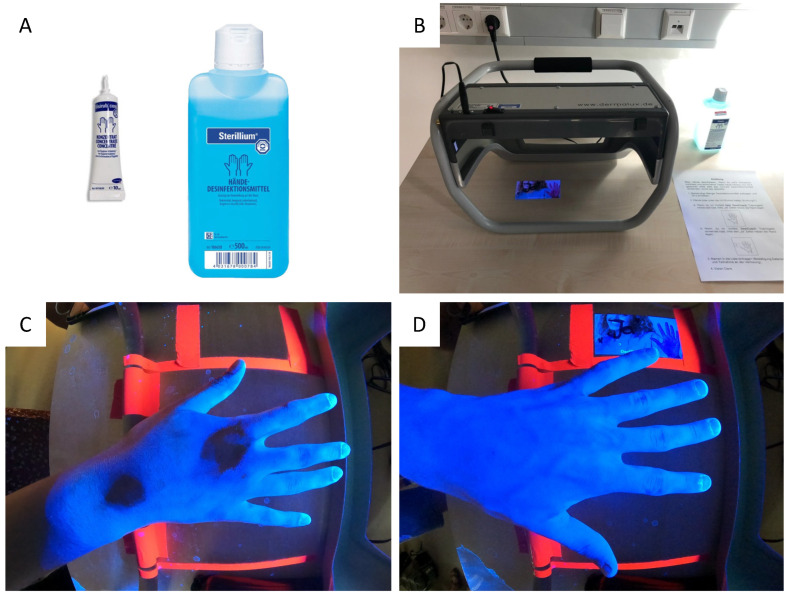
Data collection during synchronous hand disinfection with Visirub mixture (**A**), control device Dermalux Checkbox Multimedia (**B**). Exemplary depiction of a participant in cohort B1 without prior use of DesiCoach 2Go (**C**) and cohort B2 with prior use of DesiCoach 2GO (**D**).

Hand disinfection was required prior to the seminar. Hand disinfection was carried out voluntarily with Visirub instead of standard disinfectant and with a UV-visible concentrate added to an alcohol-based disinfectant (Figure 3A). Shortly before recording the results under the UV box (Dermalux Checkbox Multimedia control device, KBD GmbH, Weinheim, Germany) (Figure 3B), the participants were asked to indicate whether they had previously used DC for training (Figure 3C,D). The results of the partially or completely disinfected hands (Figure 3C,D) were recorded by using a camera (Gopro HERO Black) installed in the box, including a wide-angle lens with a 155° field of view (Gopro Max Lens Mod). The data were controlled and recorded via an iPad and the Gopro Quik app, which enabled wireless recording. For consistency and comparability with DC, the same areas as those in Figure 3B were defined and mapped in the same way. All the images taken were evaluated by the same person. The presence of the camera allowed an additional, subsequent recording of the number for the presence of foreign bodies such as watches or rings.

### 2.4. Statistics

The evaluation of the questionnaires was carried out with SPSS version 28.0 (IBM Corp., Armonk, NY, USA) using the SPSS command frequencies, descriptive statistics, a reliability analysis and a factor analysis. The results of the factor analysis were checked against Comrey and Lee’s guidelines, according to which factors greater than 0.71 were classified as “excellent”, greater than 0.63 as “very good” and greater than 0.55 as “good” [33]. Factors less than 0.55 were considered “suitable”.

The frequency analyses and distributions for the sociodemographic items as well as the responses to the hand hygiene knowledge were carried out by giving commands to the SPSS and were verified by using R language (RStudio v2022.07.2 Build 576, Posit PBC, Boston, MA, USA). The correct answer to each item was awarded with 1 point, with a maximum total score of 25. An FSU grading scheme was used to assess HH knowledge in terms of the number of points available. Very good corresponds to >90% of the answers were correct, good corresponds to 90–80%, satisfactory corresponds to 80–70%, sufficient corresponds to 70–60% and not sufficient corresponds to <60 %.

An evaluation of the untreated skin areas after DC or Visirub was performed using a descriptive analysis in Excel (v16.62). Based on the histographic distribution of the data, a Mann–Whitney U test was performed to determine the *p*-values, where a level <0.05 was rated to be significant, using R language (RStudio v2022.07.2 Build 576, Posit PBC, Boston, MA, USA).

## 3. Results

### 3.1. Survey and Participant

Of the *n* = 836 MSs and RTs contacted, *n* = 229 (27.4%) started the survey during the survey period. At the end of the survey period, *n* = 204 (24.4%) questionnaires had been fully completed. The proportion of participating individuals decreased in the further steps of the study, shown in the flowchart in Figure 1. Cohort B2 is a subset of cohort A.

The age of the respondents ranged from 18 to 44 years (mean = 24 ± 3.59). Gender was unevenly distributed in the sample, caused by the proportion of women in the health sector (male = 52; female = 152). Over 70% of the participants were residents of a large city, presumably Jena (Table 1).

### 3.2. eHealth Literacy

Regarding the question on the usefulness of the internet for personal health decisions (first additional item following Norman and Skinner) and the importance of being able to access these health resources (second additional item following Norman and Skinner), a four-point Likert scale was chosen. Because of the lack of a neutral middle, the respondents had to tend toward a positive or negative attitude in each case. Here, 71.1% chose usefulness (57.4% for useful, 13.7% for very useful, respectively) and 73% voted for the importance of being able to access these resources (45.1% for important, 27.9% for very important, respectively).

The means, internal consistency and factor analysis results of the eHEALS questionnaire are presented in Table 2. Each item uses a five-point Likert scale for response, where response options range from “strongly agree” (5) to “strongly disagree” (1). An item analysis was conducted for all items, yielding a good-fitting scale with a coefficient alpha (α) of 0.89. Item-scale correlations between items ranged from *r* = 0.58 to *r* = 0.76. A principal component analysis was conducted, which revealed a variance of 31.9 %. The factor loadings of the eight items ranged from 0.58 to 0.79.

### 3.3. Hygiene Awareness and Hand Hygiene

Of the 204 respondents, 107 (52.5%) received HH training within the calendar year of 2021. For the remaining 97 respondents, the training took place more than 1 year ago (46 %) or they had never received training (1.5%). HH training was conducted by tutors for two-thirds of the respondents. Among them, the last training of 131 (64.2%) was conducted with the UV disinfectant and that of 56 (27.5%) with normal disinfectant. When questioned whether the hand disinfectant used during the routine was an alcohol-based one, 176 (86.3%) answered yes, 18 (8.8%) answered no and 10 (4.9%) were not sure.

**Figure 4:**
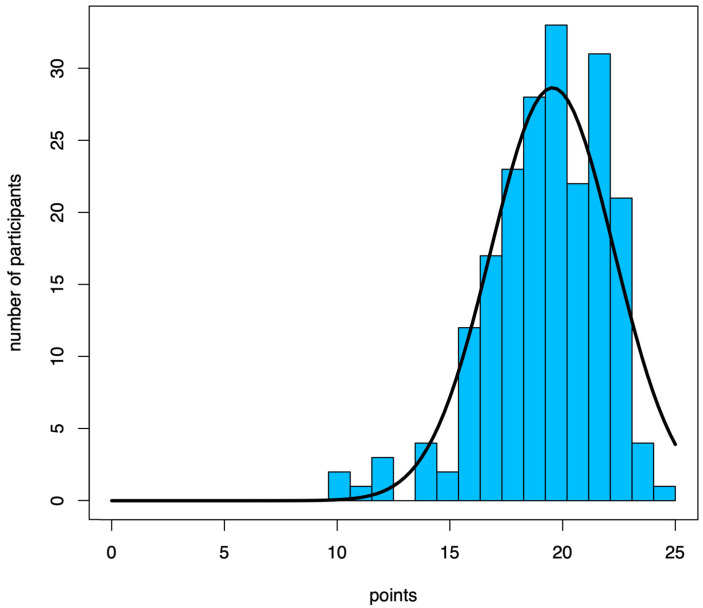
Histogram of the achieved score of the participants on the knowledge questions of the WHO questionnaire.

Next to the sociodemographic questions (results in Table 1), questions 3.5 to 3.12 of the WHO questionnaire were plotted in terms of correct score (Table 3). The 204 participants (see Figure 1) scored a mean of 19.5 ± 2.7 (median: 20, range: 10 to 25) points (Figure 4). The results of the RTs and MSs were not statistically compared against each other, because of the small size of the RT group.

According to the results of all the participants in the questionnaire, *n* = 26 (12.7%) scored “very good” (>90% correct answers), *n* = 86 (42.2%) scored “good” (90–80% correct answers), *n* = 51 (25%) scored “satisfactory” (80–70% correct answers), *n* = 31 (15.2%) scored “sufficient” (70–60% correct answers) and 10 (4.9%) scored “not sufficient” (<60% correct answers).

### 3.4. Hand Disinfection (Including Asynchronous Training)

**Figure 5:**
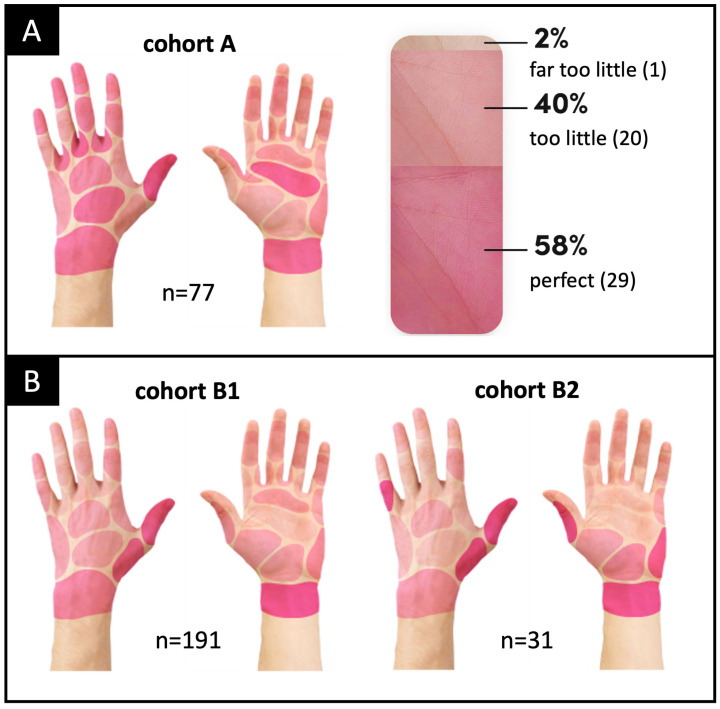
Wetting gaps of the participants by means of Heyfair frontend result presentation—the darker the area, the higher the number of participants for this wetting gap (i.e., the lack of disinfection): (**A**) shows cohort A (DC self-training) and the intensity results. (**B**) shows cohort B1 (internship participants with UV disinfectant) and cohort B2 (internship participants with UV disinfectant and previous DC self-training).

A representation of the results of DC by Heyfair are shown in Figure 5 (cohort A represents the asynchronous self-training for hand disinfection and cohorts B1 and B2 represent the synchronous hand disinfection), as pictograms with a color coding of the relative frequency of wetting failures (three color levels: <10%, 10–25% and >25%).

Cohort A includes the MSs and RTs who voluntarily decided to asynchronously participate in the testing of DC after completing the questionnaire. Cohort B comprises the voluntary participation of the documented synchronous hand disinfection with Visirub at the practical seminar. Cohort B was divided into B1, no use of DC in advance, and B2, successful performance of DC in the past.

**Figure 6:**
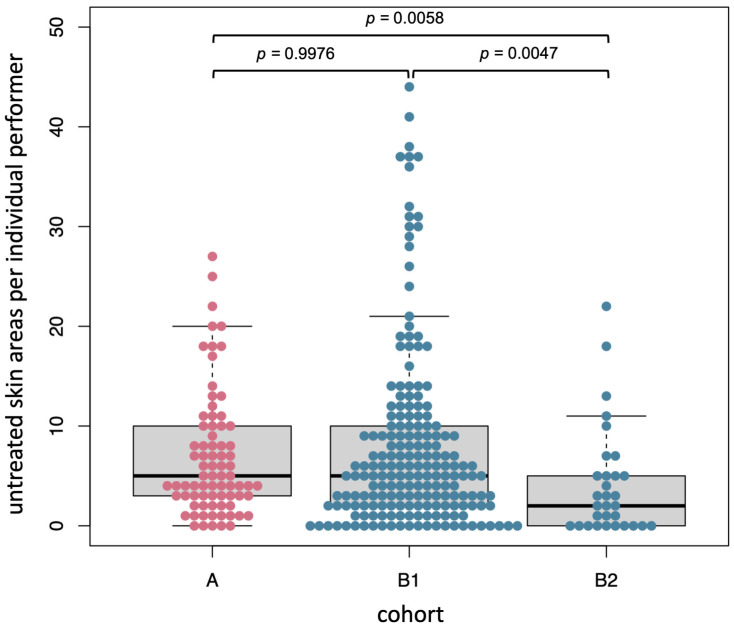
Wetting gaps of the participants by means of Heyfair frontend result presentation of cohort A (DC self-training), cohort B1 (practical seminar participants with UV disinfectant) and cohort B2 (practical seminar participants with UV disinfectant and previous DC self-training).

The visual assessment of cohort A (*n* = 77), which is based on the color gradient, shows that the back of the hand, wrist, thumb, fingers’ interdigital spaces (back) and hand line had the most wetting gaps (>25%). The mean untreated skin area was 6.8 ± 6.2 (median: 5, range: 0–27) (Figure 5A and 6). In addition, the “efficacy” was queried via the enclosed color chart, which indirectly provides information about the amount of disinfectant used. Only 60/77 participants from cohort A responded to the voluntary efficacy question (color intensity), of which only 29 (58%) indicated “perfect” staining (Figure 5A).

For cohort B1 (*n* = 191), wetting gaps were shown particularly for the thumb and the wrist. The mean untreated skin area was 8.0 ± 9.1 (median: 5, range: 0–44) (Figure 5B and Figure 6). The evaluation of the images showed that respondents often tend to overestimate themselves; in fact, 84 participants were wearing foreign objects on their wrists or fingers (82 were wearing watches or arm jewelry, 7 rings).

Cohort B2 (*n* = 31) showed the most wetting gaps for the thumb, little finger (backside) and wrist. The mean untreated skin area was 4.2 ± 5.5 (median: 2, range: 0–22) (Figure 5B and 6). The evaluation showed that eight participants were wearing foreign bodies on their wrists or fingers (7 were wearing watches and arm jewelry, 1 ring).

The comparison of the wetting gaps on the front and back of the hands of all cohorts revealed significant differences (absolute data of each area given in Supplementary Materials), except for the comparison between the front and back sides in cohort B2, as well as B1 front and A back (Table 4).

The comparison of the front and back of the hand showed significant differences in the number of wetting gaps in the individual cohorts in A (*p* ≤ 0.001) and B1 (*p* ≤ 0.001), where the back of the hand showed more wetting gaps. There was no significant difference in cohort B2 (*p* = 0.8168).

## 4. Discussion

The eHEALS questionnaire, which was introduced in 2006 and has been frequently cited since then, has proven to be an interesting instrument for assessing ehealth competence in various studies [30,34,35,36,37]. Owing to the one-point survey, it is currently not possible to make any statements about the participants’ self-assessment of ehealth competence over time. In addition, the items do not focus on the assessment of all the competence characteristics of the Lily model. However, it enables a simple, quick and straightforward analysis as an add-on to other measurement instruments or studies.

Comparable studies that observed this instrument in the past were surveys in regions with more-divergent healthcare provisions (e.g., Africa, Asia) than those in Central Europe [34,36,38]. This is only moderately suitable for comparison because here, for example, the basic prerequisites such as unrestricted access to the internet or internet access from home (13.6%) are not given [38]. At the same time, in a study by Shiferaw et al., respondents’ confidence in information found on the internet for making health decisions is not high (45.5%), with just under one-third of respondents (35.9%) stating that they have no ability to find helpful resources. This suggests that accessibility to the internet is critical for routine use and thus so is skill development in use.

Possible studies for comparison are those conducted by Holt et al. from Denmark on nurses or by Perez et al. from Spain on students of education and nursing at the University of Huelva [35,39]. A comparison with a study conducted in Cologne by Söllner et al., on the other hand, is less appropriate because this involved 12th-grade students unrelated to healthcare [40].

The respondents in the study shown here who were asked to comment on ehealth literacy were trainees and students in the healthcare sector aged between 18 and 44 years, which corresponds well with the range and gender distribution of the study by Perez et al. [35]. Our values for gender distribution are in very good agreement with the proportion of women in healthcare, at 76% [41]. Furthermore, according to a broad definition, all the participants but one (99.5%) are to be considered “digital natives”. It can therefore be assumed that the respondents with a secondary school leaving certificate or university degree have all grown up with digital end devices or had contact with them during school time. This indicates that reading and writing skills and computer competences are available in diverse forms. On the usefulness and importance of being able to access ehealth information, over 70% of the participants rated it as positive in each case. The Bertelsmann Foundation already showed in 2015 that younger adults are often more willing to engage with ehealth offers [42].

The reliability of the scale used was tested for internal consistency by using an item analysis. At 0.89, the Cronbach Alpha is highly reliable and also comparable to Perez et al.’s value, at 0.87. A comparison of the respective factors with those of Perez et al. reveals greater deviation in the question about knowing where to find useful information (Perez: 0.81 to 0.68). Nevertheless, the participants consider themselves better able to critically evaluate these sources (0.73). One possible reason may be that far fewer trainees (RTs, *n* = 13), who had so far had little training in handling digital medical databases (e.g., Pubmed), and who often learn this during their later professional life, when they have to deal with current standard operating procedures or guidelines, were interviewed. The larger proportion of respondents (MSs) had the opportunity to deal with databases and publications during their studies. Furthermore, it is not uncommon in Germany to engage with doctoral studies during medical school, which can have an additional positive impact on self-assessments for critical evaluations of sources [43].

The measurement instrument used measures only the self-perceived skills of the participants and their handling of the health information. No conclusion can be drawn on the higher-level assessability of competencies or derivable behavioral patterns, as no real skills were measured. In addition, various studies have confirmed that respondents often tend to overestimate themselves on computer-based competencies, which affects male participants more often than female participants [44,45].

With regard to a possible grading of the WHO knowledge questionnaire according on Sinopidis et al., more than half (54.9%) achieved a very good or good rating. The median and mean also showed good agreement [46]. Only 4.9% would have failed this type of single-choice test with a grade of “poor”. Other studies that used this questionnaire to assess healthcare workers’ HH showed a fairly heterogeneous but still-inferior distribution of median and mean scores [46,47,48,49]. The difference in the results presented here could be caused by the level of teaching or formal HH training, although evidence is pending. Especially in the study by Sinopidis et al. from Greece, with a matching gender distribution (*n* = 439: 27.5% male and 72.5% female), the mean and the median were both 14 (26 items), significantly worse than the mean of 19.5 and median of 20 determined here. The study by Baier et al. conducted on dental students, nursing trainees and medical-technical assistants (analogous to RTs) in Germany also showed worse results in knowledge than those presented in the study shown. In addition, a differentiation was made according to the year of training, whereby participants with a longer period of training had quantitatively given worse answers for various items (e.g., exposure time). However, one reason for this might be the current study situation, which featured a contact time for the hand disinfectant shortened by up to 15 s [50].

When asked about the correct HH measure immediately after a risk of exposure to body fluids, both groups were predominantly wrong. The differentiated teaching content of both subgroups may also be the reason for the different answers. Curricula at (vocational) schools are often closely timed, and updates in textbooks often appear only in new editions at longer intervals [51,52]. Knowledge transfer at university institutions is not infrequently practiced detached from textbooks. Exceptions such as physics, mathematics and anatomy experience similar update cycles for textbooks. Current research findings are therefore not immediately integrated into textbooks, because release processes are time-consuming [53]. However, the results from the MSs and those from the RTs were similar in most of the questions, which seems to limit the impact of this hypothesis.

A good example for recently changed evidence concerns the exposure time of the disinfectant. In numerous textbooks and information materials, at least 30 s is stated or taught. Nevertheless, studies investigated the reduced contact time of 15 s. Isolated teaching materials, including those of the Clean Hands Campaign, already proclaim these values [50]. Therefore, only 53.4% gave the correctly rated answer of at least 20 s in the questionnaire, more often by the MSs than by the RTs. It is therefore conceivable that the teaching materials of the MSs, which are updated more frequently by the medical faculty, contributed to a higher proportion of correct answers.

Although regular training is necessary for working in the healthcare sector, only 52.5% of respondents indicated that they had received practical HH training in the calendar year of 2021. Annual training is recommended by national commissions (i.e., KRINKO of the Robert Koch Institute) and is featured in internal recommendations used for certification [54]. Regarding a longer period of time (several years), almost all respondents stated that they had received training. This is also consistent with the question of who had provided the last training: the hygiene officer (23.5%), lecturer/trainer (8.3%) or tutor/student (65.7%). Almost two-thirds of respondents were trained by tutors, most likely within the university’s Skills Lab (compulsory participation at least once during the first year, then on a voluntary basis in the following years), which correlates with the question of the UV disinfectant used there (64.2%). The majority (86.3%) also knew that the hand disinfectant used in routine practice was an alcohol-based one.

During the practical session, cohort A (Figure 5), participants of the DC self-training, with a mean of 6.8 ± 6.2 or median 5.0 wetting gaps, was not significantly (*p* = 0.9967) different from cohort B1, with a mean of 8.0 ± 9.1 or median 5.0. This shows that when two groups with different verification methods were observed during their first-time use, the same results were obtained. According to feedback, no results were documented for seven recipients of the training kit. It remains unclear whether technical problems occurred (e.g., QR code not understood or instructions not read) or the motivation to participate was lost.

Cohort B2 (Figure 5), with a mean of 4.2 ± 5.5 or median 2.0 wetting gaps, showed significant improvements over itself in cohort A (*p* = 0.0058) and over cohort B1 (*p* = 0.0047). Nevertheless, the significance of these results must be viewed in a differentiated manner, as there were only a few weeks between the procedures of cohort A and those of cohort B2. At the same time, cohort B2, which is a subset of cohort A, is significantly smaller in number. However, in the feedback question, 11 participants stated that they had assessed themselves better (with fewer wetting gaps) in the past. A cognitive visual link of the mistakes made during the performance, which are individually shown by ad hoc animations in the self-training, could therefore still be a possible reason for the improvement from cohort A to cohort B2, a subgroup of the same participants (see Figure 3). Every second participant had ≤2.0 wetting gaps, which is slightly better but still comparable Gniadek et al.’s results, where 42% of the nursing students achieved a maximum of 2.0 wetting gaps out of the defined 52 [55]. Conventional training was previously often practiced only by looking at the moisture film or a third-party observation. In contrast, the two options used in this study are much better at compliance observation and allow for assessing qualitative and quantitative results, such as the information on the concentration of disinfectant. By using the three-step color chart (Figure 1A), it is possible to determine how well the prescribed amount of disinfectant was used. Currently, 3 mL is still recommended as the gold standard, although it should not fall below 2 mL [54,56]. Admittedly, a generalization of a fixed volume is questionable with regard to the strongly differentiating hand surface, but it is understandable that less surface can be wetted with reduced liquid. The color chart is therefore a good solution to determine which degree of coloring is present without analytical skills (see Lily model). The fact that only 58 % of the participants in cohort A used the recommended coloring, or the avoidably correct amount, indicates a possible deviation in the ability to estimate. When estimating liquids, various studies have reported a tendency to underestimate abilities [57,58]. Again, it was proven that almost every second person underestimated the recommended amount of disinfectant.

The implementation of the Visirub cohorts (B1 + B2) additionally showed how often foreign objects were worn on fingers and wrists despite existing knowledge in the questionnaire (avoid jewelry: 97.5%). This misbehavior was found in B1 with 84/191 (43.98%) and in B2 with 8/31 (25.81%). Similar results were reported by Szumska et al., to a comparable extent, separated by rings, bracelets and watches [59].

The heat maps of all cohorts (Figure 4) show clear wetting gaps in the thumb and wrist area, which have also been identified as problem areas in former studies [55]. Even over the period between the procedures of cohort A to those of cohort B2, no significant improvement in these areas could be achieved. A subsequent study with longitudinal observations and targeted training on the thumb and wrist areas could remedy the situation. When comparing the wetting gaps between the front and back sides of the hand, it was found that the back of the hand was almost always significantly more disinfected than the front of the hand (except B1 front to A back and B2 front to B2 back). The same had already been observed by other studies [55,60]. Possible reasons for this could be related to the amount of disinfectant, speed of implementation or quality of training. Too little disinfectant or too-slow implementation results in wetting gaps. Cohort B2 was significantly better than cohorts B1 and A at the performer level (Figure 5) and showed that the front and back of B2 were not significantly worse or better in terms of the number of wetting gaps (*p* = 0.08168). A separation between the right and left hands was not carried out, as a recording of the dominant hand in each case had not taken place.

The present study has several limitations, starting with the fact that it is a single-center study. Second, the questionnaire was conducted exclusively online, so participants with lower computer skills were excluded. Only fully answered questionnaires were considered for the evaluation, where *n* = 25 ended after answering the sociodemographic items. It can be assumed that only motivated and rather self-reflective participants gave their opinion and assessment. The first part of the questionnaire on ehealth literacy asked for a self-assessment, which was mostly possible with five answer options. Subsequently, the respondents could choose to select a neutral middle or to overestimate themselves (see all mean values of the items between “three” and “four” or “unsure” and “agree”). A correlation between actually studying the hospital presentation and the results of the WHO questionnaire were not investigated, as there was no validation of a successful review.

The practical hand disinfection was carried out during the COVID-19 pandemic, so the greater attention of the participants during the implementation of HH may be attributed to personal safety reasons. In addition, owing to time constraints, all participants were directly informed at the beginning of the practical seminar that disinfection must take place. The students were informed about the possibility to support the study, which led to a high number of volunteers. For this reason, the results might be biased in that the presence of Visirub led to above-average attempts to practice (in accordance with the Hawthorne effect). A link between the individually given answers to the knowledge questions and the practical hand disinfection carried out in each case was not investigated, which would have enabled evaluations about result behavior patterns (knowledge, ability, competences). From a data protection point of view, the information was recorded anonymously with DC, so that this point could not have been realized. A correlation between the success of disinfection and the used rub-in technique was not investigated, but it might have allowed insights into the causes of failure in certain areas [61].

The photo documentation was evaluated by only one person, owing to time constraints. During the evaluation, the documentation made obvious which participant had been assigned to group B1 or group B2. In addition, only wetting gaps or areas were quantitatively considered. A differentiation according to the size of the wetting gaps (pixel-by-pixel evaluation), as in the study by Fichtner et al., was not applied in our study. Because the hand disinfection training in cohort A was conducted with DC, the participants would have had to additionally carry out photo documentation themselves to allow such conclusions and comparisons [60].

## 5. Conclusions

The implementation of digital ehealth and training applications requires a certain level of competence to use and understand them. Even in a generation of “digital natives”, these skills are not developed equally by all. On average, study participants rated their eHL as sufficient for finding, using and critically appraising health information. The majority indicated that the internet helps them make health decisions and is essential for accessing health resources. Knowledge of hand disinfection according to the WHO questionnaire showed above-average results, where only 2/26 items were answered predominantly incorrectly. This may be due to a high level of hygiene education and training. Even though theoretical knowledge is not synonymous with correct implementation, the presented study showed satisfactory hand disinfection (average < 10% wetting gaps) in all cohorts. The cohort that had self-educated through asynchronous handrub training (with temporary visible disinfectant) significantly improved when UV disinfectant was applied unannounced. The back of the hand was significantly more disinfected in two out of three cohorts. In summary, it is feasible to implement data-protection-compliant, asynchronous, digital hand disinfection training (e.g., with DC). However, it is more difficult to ensure a level of willingness to participate in asynchronous training that is similar to the level of willingness to participate in synchronous training (e.g., with Visirub).

## Figures and Tables

**Table 1:** Characteristics of the respondents.

Variable	Result
Age [mean ± SD (median; range: min–max)]	24 ± 3.59 (23; 18–44)
Gender	
Female	152 (74.5%)
Male	52 (25.5%);
Place of residence	
Large city (>100,000 inhabitants)	146 (71.6%)
Medium-size city (>20,000 inhabitants)	20 (9.8%)
Small town (>5000 inhabitants)	18 (8.8%)
Rural area (<5000 inhabitants)	20 (9.8%)
Last educational qualification	
Intermediate school leaving certificate	9 (4.4%)
Qualification for university entrance	148 (72.5%)
University degree	36 (17.6%)
Completed vocational training	11 (5.4%)
Current status	
In training for radiology technician	13 (6.4%)
In medical studies (university)	191 (93.6%)

**Table 2:** The eHEALS scale mean self-assessment, reliability and factor analysis.

Item	Mean ± SD	Factor Loading	Mean Item-Total Correlation
I know what health resources are available on the internet	3.35 ± 0.98	0.71	0.72
I know where to find helpful health resources on the internet	3.69 ± 0.92	0.79	0.76
I know how to find helpful health resources on the internet	3.76 ± 0.92	0.68	0.69
I know how to use the internet to answer my questions about health	3.84 ± 0.91	0.76	0.73
I know how to use the health information I find on the internet to help me	3.86 ± 0.95	0.65	0.72
I have the skills I need to evaluate the health resources I find on the internet	3.90 ± 0.93	0.73	0.58
I can tell high-quality health resources from low-quality health resources on the internet	3.82 ± 0.92	0.77	0.60
I feel confident in using information from the internet to make health decisions	3.54 ± 0.92	0.58	0.60
Variance accounted for = 31.9%			
Coefficient alpha = 0.89			

**Table 3:** Hand hygiene (HH) knowledge of radiology technician trainees (RTs) and medical students (MSs).

Knowledge Statements *(Correct Responses)*	RT, *n* (%)	MS, *n* (%)	Total, *n* (%)
Which of the following is the main route of the transmission of potentially harmful germs between patients? *(Healthcare workers hands when not clean)*	5 (38.5)	173 (90.6)	178 (87.3)
What is the most frequent source of germs responsible for healthcare associated infections? *(Germs already present on or within the patient)*	10 (76.9)	117 (61.3)	127 (62.3)
Which of the following hand hygiene actions prevents the transmission of germs to a patient?			
Before touching the patient *(yes)*	13 (100)	189 (99)	202 (99)
Immediately after a risk of body fluid exposure *(no)*	1 (7.7)	62 (32.5)	63 (30.9)
After exposure to the immediate surroundings of a patient *(no)*	7 (53.8)	70 (36.6)	77 (37.7)
Immediately before a clean/aseptic procedure *(yes)*	11 (84.6)	188 (98.4)	199 (97.5)
Which of the following hand hygiene actions prevents the transmission of germs to healthcare workers?			
After touching a patient *(yes)*	13 (100)	189 (99)	202 (99)
Immediately after a risk of body fluid exposure *(yes)*	13 (100)	190 (99.5)	203 (99.5)
Immediately before a clean/aseptic procedure *(no)*	3 (23.1)	116 (60.7)	119 (58.3)
After exposure to the immediate surroundings of a patient *(yes)*	11 (84.6)	186 (97.4)	197 (96.6)
Which of the following statements on alcohol-based handrubbing and handwashing with soap and water are true?			
Handrubbing is more rapid for hand cleansing than handwashing *(true)*	4 (30.8)	115 (60.2)	119 (58.3)
Handrubbing causes skin dryness more than handwashing *(false)*	3 (23.1)	162 (84.8)	165 (80.9)
Handrubbing is more effective against germs than handwashing *(true)*	11 (84.6)	149 (78)	160 (78.4)
Handwashing and handrubbing are recommended to be performed in sequence *(false)*	3 (23.1)	102 (53.4)	105 (51.5)
What is the minimal time needed for alcohol-based handrub to kill most germs on your hands? *(20 s)*	3 (23.1)	106 (55.5)	109 (53.4)
Which type of hand hygiene method is required in the following situations?			
Before palpation of the abdomen *(rubbing)*	10 (76.9)	176 (92.1)	186 (91.2)
Before giving an injection *(rubbing)*	10 (76.9)	187 (97.9)	197 (96.6)
After emptying a bedpan *(rubbing)*	8 (61.5)	124 (64.9)	132 (64.7)
After removing examination gloves *(rubbing)*	6 (46.2)	171 (89.5)	177 (86.8)
After making a patient’s bed *(rubbing)*	9 (69.2)	165 (86.4)	174 (85.3)
After visible exposure to blood *(washing)*	6 (46.2)	103 (53.9)	109 (53.4)
Which of the following should be avoided, as it is associated with an increased likelihood that harmful germs will colonize on hands?			
Jewelry *(yes)*	9 (69.2)	190 (99.5)	199 (97.5)
Damaged skin *(yes)*	10 (76.9)	187 (97.9)	197 (96.6)
Artificial fingernails *(yes)*	9 (69.2)	189 (99)	198 (97.1)
Regular use of a hand cream *(no)*	9 (69.2)	184 (96.3)	193 (94.6)

**Table 4:** Comparison between back of hand and front of hand of all cohorts (in relation to the total number of wetting gaps).

	A Back	B1 Back	B2 Back
**A front**	<0.001	<0.001	<0.001
**B1 front**	0.5037	<0.001	<0.001
**B2 front**	<0.001	<0.001	0.8168

## Data Availability

Data for wetting gaps (cohort A, B1 and B2) are given in the Supplementary Data.

## Supplementary Materials

The following supporting information can be downloaded at: https://www.mdpi.com/article/10.3390/healthcare11101475/s1, Figure S1: Number of wetting gaps for all areas (front and back of hand) and cohorts.
