# Supplementary material for: Medical Students’ and Radiology Technician Trainees’ eHealth Literacy and Hygiene Awareness—Asynchronous and Synchronous Digital Hand Hygiene Training in a Single-Center Trial
Source: Healthcare (Basel). 2023 May 18;11(10):1475. doi: 10.3390/healthcare11101475 (PMC10218341; doi:10.3390/healthcare11101475)
Supplement: Supplementary file 1 [file healthcare-11-01475-s001.zip › healthcare-2274832-supplementary (1).pdf]

Number of wetting gaps for all areas (front and back of hand) and cohorts:

Front of hand

CODE	A	B1	B2
**RF1mid4**	22	3	0
**RF1base1**	4	17	1
**RF1base2**	4	11	1
**RF1base3**	7	10	1
**RF1base4**	5	7	1
**RF1gap1**	6	7	0
**RF1gap2**	9	21	1
**RF1gap3**	8	15	1
**RF1mid1**	3	16	2
**RF1mid2**	7	12	0
**RF1mid3**	3	3	0
**RF1mid5**	2	30	4
**RF1mid6**	2	16	3
**RF1thumb1**	6	0	0
**RF1thumb2**	1	13	5
**RF1thumb3**	1	0	0
**RF1tips1**	0	6	1
**RF1tips2**	0	0	0
**RF1tips3**	0	0	0
**RF1tips4**	0	0	0
**RF1wrist1**	2	30	5
**RF1wrist2**	9	125	10
**LF1mid4**	17	6	0
**LF1base1**	8	10	1
**LF1base2**	7	11	0
**LF1base3**	7	15	1
**LF1base4**	9	12	1
**LF1gap1**	6	4	0
**LF1gap2**	6	15	1
**LF1gap3**	6	20	0
**LF1mid1**	3	3	1
**LF1mid2**	7	19	0
**LF1mid3**	3	5	1
**LF1mid5**	9	16	6
**LF1mid6**	4	15	1
**LF1thumb1**	6	1	0
**LF1thumb2**	3	19	6
**LF1thumb3**	2	5	1
**LF1tips1**	1	1	0
**LF1tips2**	1	0	0
**LF1tips3**	1	0	0
**LF1tips4**	1	1	0
**LF1wrist1**	2	17	1
**LF1wrist2**	12	129	9

Back of hand

CODE	A	B1	B2
**LB1base1**	7	24	8
**LB1base2**	5	14	0
**LB1base4**	9	23	0
**LB1base3**	6	14	0
**LB1gap1**	8	6	0
**LB1gap2**	14	10	0
**LB1gap3**	15	11	0
**LB1mid1**	5	24	1
**LB1mid2**	14	17	2
**LB1mid3**	6	11	0
**LB1mid4**	13	22	0
**LB1mid5**	5	73	11
**LB1thumb1**	12	66	8
**LB1tips1**	8	23	0
**LB1tips2**	9	12	1
**LB1tips3**	6	16	0
**LB1tips4**	7	21	0
**LB1wrist1**	15	72	2
**RB1base1**	4	21	3
**RB1base2**	7	9	0
**RB1base4**	7	24	3
**RB1base3**	13	15	0
**RB1gap1**	12	6	0
**RB1gap2**	12	8	0
**RB1gap3**	14	8	0
**RB1mid1**	4	14	1
**RB1mid2**	8	18	1
**RB1mid3**	7	20	1
**RB1mid4**	4	25	1
**RB1mid5**	4	55	7
**RB1thumb1**	14	59	7
**RB1tips1**	4	16	1
**RB1tips2**	4	18	1
**RB1tips3**	5	9	1
**RB1tips4**	6	20	1
**RB1wrist1**	12	57	3

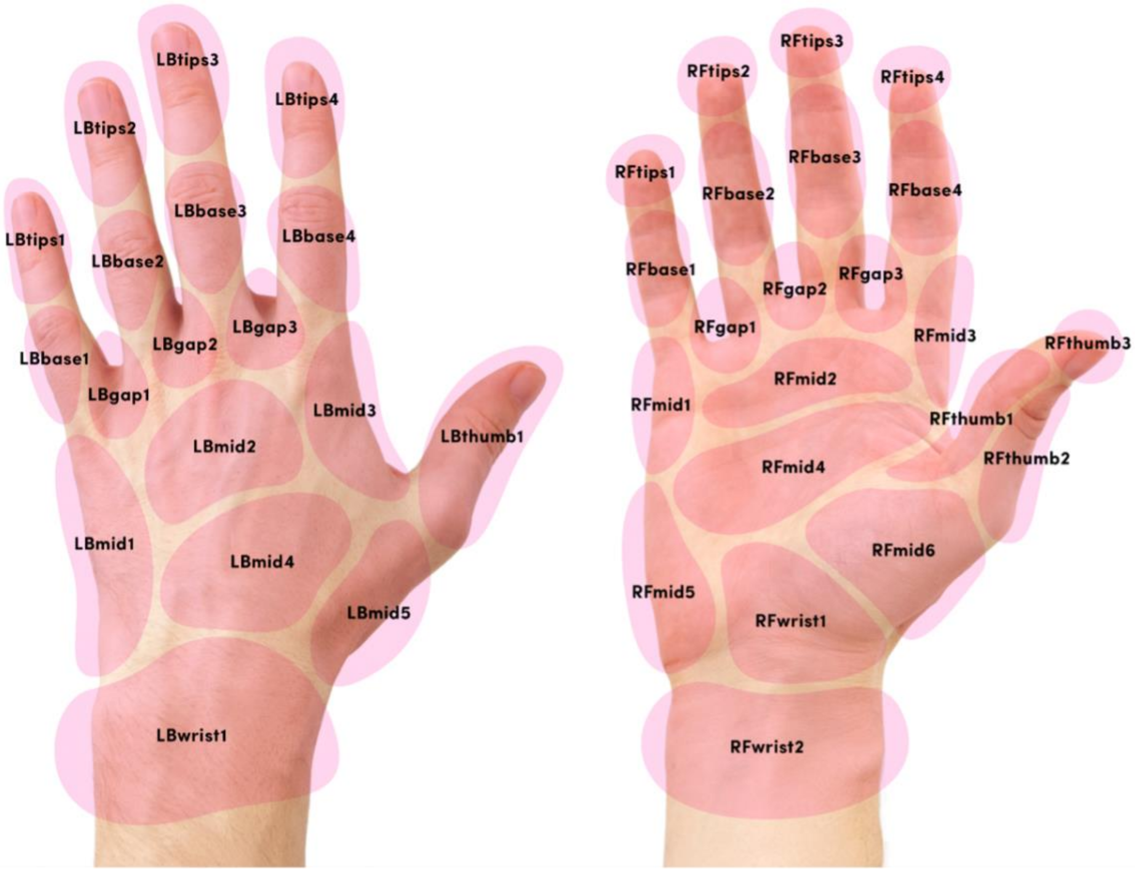
LBtips3
LBtips2
LBtips4
LBbase3
LBtips1
LBbase2
LBbase4
LBgap3
LBgap2
LBbase1
LBgap1
LBmid3
LBmid2
LBthumb1
LBmid1
LBmid4
LBmid5
LBwrist1
RFtips3
RFtips2
RFtips4
RFtips1
RFbase3
RFbase2
RFbase4
RFbase1
RFgap2
RFgap3
RFgap1
RFmid3
RFthumb3
RFmid2
RFmid1
RFthumb1
RFmid4
RFthumb2
RFmid6
RFmid5
RFwrist1
RFwrist2
